# Sport‐specific differences in ACL injury, treatment and return to sports: Football

**DOI:** 10.1002/ksa.12803

**Published:** 2025-08-15

**Authors:** Werner Krutsch, Dominik Szymski, Johannes Rüther, Volker Musahl, Alberto Grassi, Thomas Tischer, Markus Gesslein

**Affiliations:** ^1^ SportDocsFranken Nürnberg Germany; ^2^ Department of Trauma Surgery University Medical Center Regensburg Regensburg Germany; ^3^ Department of Orthopedics and Traumatology Paracelsus Medical University Nürnberg Germany; ^4^ Department of Orthopaedic Surgery, UPMC Freddie Fu Sports Medicine Center University of Pittsburgh Medical Center Pittsburgh Pennsylvania USA; ^5^ II Clinica Ortopedica e Traumatologica, IRCCS Istituto Ortopedico Rizzoli Bologna Italy; ^6^ Clinic for Orthopaedics and Trauma Surgery, Malteser Waldkrankenhaus St. Marien Erlangen Germany; ^7^ Department of Orthopaedic Surgery University of Rostock Rostock Germany

**Keywords:** ACL rehabilitation, ACL surgery, amateur, knee injury, prevention programme, professional, soccer

## Abstract

The management of anterior cruciate ligament (ACL) ruptures is carried out differently in prevention, treatment and rehabilitation for athletes than for non‐athletes. However, different approaches to managing ACL ruptures are also discussed in different sports. This narrative review includes first time the current situation of ACL management in football by experiences from the practical routine as well as from scientific reports. No other sport shows such high number of literature reports and sufficient scientific evidence in the management of ACL ruptures like football and this article provides a football specific overview in prevention, treatment and return to sports strategies. These scientific reports underline the surgical treatment of ACL ruptures in football player and show a high sustainability and return to sports rate. However, reports from football show that despite good knowledge, the implementation of sustainable prevention and rehabilitation is difficult and needs improvement.

**Clinical Trial Registration:**

Considering that this manuscript is a narrative review, no clinical trial registration is neccesary.

**Level of Evidence:**

Level V.

**Abbreviations**
ACL: anterior cruciate ligament
AP: anterior posterior
BTB: bone‐tendon‐bone
FIFA: Federation Internationale de Football Association
HT: hamstring tendon
HIIT: High Intensity Interval Training
QT: quadriceps tendon
LET: lateral extra‐articular tenodesis
PEEK: polyetheretherketon
PT: patellar tendon
UEFA: Union of European Football Associations

## INTRODUCTION

In professional as well as in amateur football, Anterior cruciate ligament injuries (ACL) can lead to extended periods of absence from the sport, impacting not only player performance but also team dynamics and financial aspects of the club [35]. Arthroscopically assisted knee surgery in athletes has undergone further developments in recent years [30]. In football, special developments have been driven by FIFA, UEFA and national football organisations for over 20 years and include educational programmes for medical professions such as UEFA's Football Doctor Education Programme and FIFA's Diploma in Football Medicine. In addition to a large number of specialist books on football medicine [15, 22, 38], there are also many scientific reports on various topics of football medicine, which includes especially topic about prevention or treatment in ACL injuries [3, 5, 32, 44, 47]. Therefore, it is crucial to better understand the sport‐specific ACL injury mechanisms to optimise the treatment and to develop prevention strategies [45, 47]. This narrative review provides for the first time sport‐specific information in the management of ACL injuries in football, including both scientific evidence and literature reports from practical routine. Taking into account a large number of scientific publications on football and also about ACL ruptures [4, 9, 37], this article presents the various topics such as prevention, therapy and return to play procedures according to their evidence and practicality exclusively for football. The main objective of this work is to present the different aspects in the management of ACL ruptures for different professional groups in football such as physicians, physiotherapists and coaches.

## INJURY MECHANISM OF ACL INJURIES IN FOOTBALL

According to the emerging evidence on video analysis, ACL rupture in footballers typically is a non‐contact, decelerating and defensive injury. Overall, recent studies found that mechanisms of ACL injury in football can be categorised into three distinct types: non‐contact, indirect contact and direct contact [11]. Non‐contact injuries in football are defined as those occurring without any contact to the knee or other parts of the body prior to the injury frame. On the other hand, a certain proportion of ACL ruptures are also caused by contact injuries, which are sometimes caused by fouls, but much more often by typical game movements. Such contact injuries can occur as direct contact to the injured knee joint or as indirect contact to the player's upper body, thus acting as disruptive factors in the accident mechanism during the landing of the leg. Current research has pointed out that many ACL injuries in professional male football occur without direct contact [33]. According to the findings, up to 88% of ACL injuries were identified during video analysis as occurring without direct knee contact [11]. Notably, 44% of these injuries were attributed to indirect contact, predominantly involving interactions at the upper body or pelvis level. In principle, all injury mechanisms for ACL ruptures are part of normal football play and are rarely sanctionable. In terms of biomechanics, the kinematics or intersegmental relationships between body segments during the initial contact phase and actual injury are crucial to understanding ACL injuries. For instance, during high‐intensity play, the need for rapid deceleration or changes in direction increases the risk of non‐contact injuries. This is particularly evident close to the side‐lines and in the penalty area, where in fact most of the injury occurs. These moments often coincide with dynamic situations such as pressing, where players experience deceleration forces that can compromise knee stability. These forces can be magnified when football players engage in high‐speed movements with unstable foot placement. Therefore, playing on unexpected pitch surfaces can further increase the risk of ACL injuries due to inability to maintain optimal traction which can lead to missteps or erratic movements [13].

Football‐specific research has recently found that women's football has other specific injury mechanisms, which for the first time can be divided into four categories [1]. Beside the well‐known non‐contact injury in ‘pressing situation' and the indirect contact ‘parallel to sprinting and tackling', did the authors also found direct contact mechanism ‘knee‐to‐knee' and the non‐contact ‘landing mechansim'. Another emerging football‐specific concept related to ACL injury is the role of neurocognitive errors. In fact, nearly half of non‐contact injuries has been somewhat related to poor decision‐making when approaching the opponent with high speed and with reduced ability to stop and change the intended action, or to attentional errors with player shifting his selective attention away from the relevant task to irrelevant stimuli [21].

Current literature consistently demonstrates that ACL injuries in football predominantly manifest during early knee flexion accompanied by dynamic knee valgus loading. Some studies, indicate a prevalent knee‐dominant movement pattern. This pattern involves limited loading and mobility in joints other than the knee, suggesting a focused transfer of forces to the knee joint that may predispose to ACL injuries [49]. Certain hip and knee flexion angles significantly increase the risk of ACL injury. Especially when a player suddenly pivots or lands from a jump, an excessive valgus angle at the knee with consecutive collapse inward can occur with an increased hip motion [3]. Such frontal plane motion is potentially linked to the external knee abduction moments that arise from hip abduction forces. Notably, in many cases knee valgus loading can be prevalent without valgus collapse, indicating that while knee valgus is common, severe forms of it might be less frequent. On video assessment of sagittal plane mechanics at initial contact, data show football players often maintain an upright trunk position with notable hip flexion and slight knee flexion. Nearly half demonstrated early plantar flexion at the ankle during heel strike, highlighting specific biomechanical configurations that may influence ACL injury susceptibility [11]. The role of muscle activation patterns including the hamstrings and quadriceps muscles is crucial. Due to the inherent sprinting and cutting movements in football a proper balance between these muscle groups can help stabilise the knee. However, muscle fatigue may disrupt these activation patterns, leaving the ACL more vulnerable during explosive actions such as sprinting or rapid directional changes [3]. The situational patterns associated with ACL injuries in football can generally be divided into defensive and offensive situations. An injury resulting from a defensive action, such as a tackle, may arise from an opponent's approach with the intent to disrupt a play. Some studies highlight that non‐contact injuries may more often occur to players in high‐pressure situations when attempting to make a crucial tackle due to the elevated intensity and urgency of the actions involved. Therefore, defenders may be more susceptible due to their frequent need to react quickly to an opponent's unpredictable movements, leading to unexpected forces being applied to the knee joint [23].

## INJURY PREVENTION STRATEGIES OF ACL INJURIES IN FOOTBALL

Successful primary injury prevention begins with the analysis and understanding of the injury mechanisms in each sport, but knowledge of sport‐specific influencing factors also plays an important role [2]. Various epidemiological studies from international amateur and professional football show typical football‐related risk factors for both women's and men's football. A previous injury to the ipsilateral or contralateral knee joint or minor injuries to the lower extremities overall are not purely football‐specific risk factors for an ACL rupture, although these are considered as the most important risk factors for the occurrence of an ACL ruptures in football [47]. Typical football‐related factors include a short‐term change of playing level, a change of coaches or other reasons that lead to he need for neuromotor adaptation in football player [33, 41]. A gradual adaptation and habituation of the players to short‐term changes and especially to short‐term increasing physical demands are part of the successful prevention of ACL ruptures in football teams, which must be continually adjusted over the course of a season.

Highest evidence in the primary prevention of sports injuries shows the implementation of injury prevention programmes in training or warm up routine [4]. Injury prevention programmes have been implemented and published in large numbers, particularly in football. Such prevention programmes have also been implemented in football to prevent ACL ruptures and have shown very good results. The well‐known and general injury prevention programme FIFA 11+ was able to reduce also ACL ruptures in amateur football [42]. For structural reasons, such studies and programmes are rarely implemented in professional football. Thus, one study in Germany did examine the evidence of a prevention programme in salaried semi‐professional elite football. The results showed that regular implementation of the 5 exercise modules per training session took no more than 10 min, but the reduction in serious knee joint injuries like ACL ruptures was more than 50% compared to the control group without the programme. The programme includes 5 training modules for a football training to reduce these knee injuries [31]:

I) *Mobility preparation of joints and muscles*
II) *Trunk stability exercises*
III) *Leg axis stability exercises*
IV) *Jumping and landing exercises*
V) *Multidirectional agility training*

General concepts for preventing knee injuries in football have already been published in various ways. Although these concepts have little evidence in terms of their overall sustainability, they include both evidence‐based parts of successful prevention programmes and also a football‐typical approach to implementation in practical everyday football routine. Krutsch and Loose published in 2020 eight different prevention strategies for clubs in elite football [31]:

I) *Analysis of deficits in currently used prevention concepts*
II) *Epidemiological injury analysis about incidence and risk factors*
III) *Implementation of injury prevention training programmes*
IV) *Video analysis for risk factors and injury mechanism*
V) *Neuromotorical screening tests to identify deficits in players*
VI) *Analysis and adaptation of current rehab strategies*
VII) *Analysis and adaptation of return to play strategies after injuries*
VIII) *Psychological (preseason) screening to identify mental status*

When using prevention programmes, it should be noted that they have rarely been studied solely for the prevention of ACL ruptures. However, it is also pragmatically correct that the prevention programmes used in football not only prevent one type of injury, but also apply to a variety of injuries to improve player participation [31]. In addition to primary injury prevention, there is also secondary and tertiary injury prevention in football. These types of injury prevention involve the earliest possible detection of previously occurred ACL ruptures and the most optimal treatment of the injury [38]. The goal of secondary and tertiary prevention of ACL ruptures in football is to prevent recurrent injuries and several publications specifically for ACL injuries in football are available [16, 47].

## FOOTBALL‐SPECIFIC ASPECTS OF NON‐SURGICAL TREATMENT OF ACL INJURIES

In general, and for all populations of athletes and non‐athletes, systematic reviews also show the possibility of non‐operative treatment of ACL ruptures [20, 29], while information specifically for the football playing population show a trend to improved knee function and safe return to football after surgical repair. A recently published study by the national obligatory trauma insurance for professional sports in Germany showed that in over 200 ACL ruptures in professional sports, not a single ACL rupture was attempted to be treated non‐surgically [5]. Restoring stability in the injured knee joint of a football player, which was caused by the ACL rupture or possible accompanying injuries, is the ultimate goal of treatment. Since such stability cannot be achieved without surgical stabilisation in the case of complete ruptures or high partial ruptures of the ACL, surgical reconstruction of stability is recommended in sports such as football with its high pivoting movements during play [30].

Considering that torn ACL fibres do not automatically return to the original attachment of the ACL, it must be assumed that the stability that has developed after a partial rupture of the ACL will not relevantly improve further over the rehab. Clinical testing of AP stability and rotational stability can therefore be used to decide early after trauma on whether a minimal partial rupture (e.g., less than 20% torn fibres) does not lead to any relevant instability and can therefore be treated without surgical reconstruction. Another important aspect to remark on is the extremely high burden of meniscal injuries concomitant to ACL rupture. This is due to the high compressive forces and axial load during single‐leg deceleration, coupled with rotations and translations typical of the football‐specific injury [28]. A clinical study conducted on a specific population of professional footballers reported an 86% rate of concomitant meniscal injuries. Moreover, the rate of medial meniscal injuries (73%) and lateral root tears (33%) were significantly higher than in professional skiers, where no lateral root tears were reported at all [18]. In footballers it is thus common to deal with specific combinations of ACL, root and ramp tears in a relevant number of cases, more than in other sports with different motor tasks and injury mechanisms such as ski. In such cases, conservative therapy not only involves avoiding concurrent injuries to the cartilage or meniscus, but also allowing a healing and rehabilitation period of 2‐3 months after trauma to prevent subsequent injuries.

After deciding on conservative treatment for a partial ACL rupture, rehabilitation should include both a gradual build‐up and integration into football and also sport‐specific tests of neuromotor skills and leg axis stability. These motor skills can be tested using a functional test battery and are widely known since the last decade as return‐to‐play tests after surgical treatment of ACL ruptures [25]. The rehabilitation of non‐surgically treated ACL ruptures shows specifically for football players no clear evidence about weight‐bearing, bracing and restricted range of motion in the early phase and is also missing in the practical routine due to rare cases. Such tests are widely carried out as return‐to‐play tests after surgical ACL reconstruction, but can also be carried out before returning to the field to ensure a treatment decision for conservative therapy. Since professional players always tries to improve the chances of recovery and shorten the time it takes to return to the field, additional measures are always used to improve conservative therapy or rehabilitation after an ACL rupture.

Non‐surgical treatment of ACL ruptures in football is generally restricted to indications such as minimal partial ACL ruptures, players who wish to avoid adequate stability, and thus often those football players who intend to retire. Specific conservative treatment methods for ACL ruptures with a certain degree of evidence do not exist, at most case reports without football‐relation. According to current literature, measures such as shock waves, intra‐articular injections with orthobiologics or similar procedures show several reports in the literature but no evidence in football and are therefore not generally recommended for football players with ACL injuries [26].

## FOOTBALL‐SPECIFIC ASPECTS OF SURGICAL TREATMENT OF ACL INJURIES

ACL reconstruction in football players must restore a normal anatomical knee function to compensate the substantial impact on the knee joint during a game. The challenge consists in a shift between the fastest possible return to play and the specific risk of re‐rupture. Specific intraoperative aspects should be considered in football players, including graft choice, concomitant joint pathologies, extra‐articular supplementary stabilisations, and graft fixation methods.

Selection of an appropriate graft type and size is an important decision that impacts the overall success of ACL reconstruction. A recently published consensus of international knee surgeon and sports physician revealed no consensus regarding importance of graft choice in return to football [19]. With increasing professional level primary stability after surgery becomes more important for the player to pass through an accelerated, standardised rehabilitation programme back to his pre‐injury level. The two primary types of grafts are autografts and allografts. Autografts include options such as the patellar tendon (PT), quadriceps tendon (QT) or hamstring tendon (HT). Autograft selection depends on various factors, including the patient's age, professional sports level, and personal preferences [14]. In the last decade the hamstring tendon has gained further preference due to its minimal donor site morbidity compared to the patellar tendon, which was traditionally viewed as the gold standard in former times. However, the PT remains popular among professional football players due to its inherent strength, its tensile properties and the possibility to harvest a bone‐tendon‐bone (BTB) transplant. Lower Re‐rupture in BTB autografts have also been demonstrated across various sports in young players including football [14]. Despite those data and an assumed faster osteointegration of BTB transplants, the surgeon must consider the higher rate of intra‐ and postoperative complications and donor site morbidity associated with PT autografts such as patella fracture and persistent anterior knee pain [34]. On the other hand, graft choice must also consider the specific demands of football, as players frequently require rapid bursts of speed and quick lateral movements. However, the donor site morbidity of hamstring harvesting tendons and the impairment on muscle kinematics is usually well tolerated by footballers and does not seem to alter the sport performance, which is determined by a complex interplay between physical capacity, technical skills and team‐work. A recent meta‐analysis compared the isokinetic strength of the muscular knee joint between QT, HT or PT autografts after ACL reconstruction [24]. The QT showed significant better results with knee flexion compared with HT and similar results to PT at 6 and 12 months. While HT showed better and significant results with knee extension at 6 months and similar results at 12 months compared to QT. Concerns exist regarding the long‐term outcomes of allografts, particularly in high‐demand athletes like in football. For instance, a more recent investigation found a higher re‐injury rate in athletes using allografts compared to those with autografts [8]. This has led to a consensus that autografts may offer superior outcomes in young athletes engaged in high‐impact sports like football. In the recently published study on ACL ruptures in German professional sports, the use of HT as grafts was found in over 70% of all professional sports. Only in professional football were PT and QT used more frequently in nearly half of all cases. The reason for this fact is not so much the scientific background ACL treatment in football as the choice of surgeons and their preferred choice of graft [5].

Most important aspect of fixation is the positioning of the graft within the anatomical landmarks. Only accurate anatomical placement and tensioning will restore natural knee biomechanics. The method of graft fixation within the anatomical landmarks is critical in promoting the integration of the graft and ensuring its longevity, but most commonly used fixation devices have shown sufficient primary stability for football players [10]. The two most popular fixations methods in football players are interference screws (biodegradable or metal) and suspensory fixation devices (e.g., cortical buttons). The choice between screws can depend on factors such as the graft type and the surgeon's preference. However, in a recent study bioabsorbable screws were associated with a significantly higher risk of complications, such as graft rupture, joint effusion, and infection [48]. While non‐absorbable screw fixation (PEEK or metal) provides a reliable and long‐lasting stability, removal during revision surgery can be demanding. Recent advancements in fixation devices have led to a shift towards suspensory fixation systems, particularly for HT grafts in amateur players as well as professional players [6]. Modern adjustable suspensory fixation need can provide direct tendon‐to‐bone healing without implants, but require a good fit between graft and the drill tunnel, which can be achieved using the press‐fit technique. This results in improved graft tensioning capabilities and stability post‐surgery also for QT grafts [10]. The trend that surgeons use hybrid fixations for highly demanding professional footballers is not based on scientific evidence and rather on the surgeon's selection.

Research indicates that the inclusion of Lateral Extra‐articular Tenodesis (LET) in ACL reconstruction surgery can significantly improve stability, particularly in patients exhibiting signs of rotational laxity. This may be particularly relevant in football players, where rapid pivoting and twisting movements are common. The addition of LET reduced the risk of undergoing revision by 2.8 times in elite athletes undergoing primary ACL reconstruction and the risk reduction did not differ significantly between the patellar tendon and hamstring tendon autografts [7]. Athletes receiving an ACL reconstruction with the addition of a LET procedure were 2.42 times more likely to return to preinjury performance at 2 years than athletes with ACL reconstruction alone [39]. LET is described as not significantly associated with prolonged RTP. However, the need for extra‐articular stabilisation should be balanced against potential complications because of the additional surgical time, additional skin incisions, additional use of external material and their typical consequences. An important factor for the use of LET is the evaluation of individual factors for a potential re‐rupture, which can be rotational instability, general hyperlaxity or, for example, a high tibial slope. Surgeons must consider patient‐specific factors and the potential for morbidity associated with additional surgical procedures like restricted range of motion and revision rate. A tailored approach ensures optimal functionality, especially for professional football players where the stakes are high [17].

## FOOTBALL‐SPECIFIC REHABILITATION AND RETURN TO SPORTS AFTER ACL INJURIES

Scientific reports on the rehabilitation of ACL ruptures are widespread, but rarely focus on a sports‐specific approach [36, 46]. As football involves sudden pivots, landings, and changes in direction, the rehabilitation of ACL injuries requires an integrated approach combining physiological, psychological, and sport‐specific training methodologies. The initial injury mechanism should be paid special attention as injuries frequently occur during non‐contact or indirect contact situations. Excessive force loading on the knee is often caused by disbalances in the pelvis or asymmetric force distribution within the hips and ankle joints. Therefore, concomitant pathological movement patterns need to be identified in ACL injured football player and addressed during the rehabilitation process. Football‐specific rehabilitation and the sport‐specific return to football field is well‐described in many publications and includes many football‐specific aspects [38].

The early rehab period of the first 12 weeks after surgery is not in detail sport‐specific and includes acute phase, early rehab phase and functional rehab phase. In addition to the control of possible postoperative symptoms, this phase includes the general restoration of mobility, musculature and balancing ability. Cardiovascular training, strength training and initial athletic skills are the main focus here. The rehabilitation period between 4 and 6 months includes the first individual football‐specific exercises, where the strength, proprioception, athletics, neuromotor and cardiovascular skills are improved. Special consideration should be given to graft specific loss of strength, as patellar or quadriceps grafts will lead to prolonged recovery of extension strength while hamstring grafts will show reduced flexion strength. After 6 months following ACL surgery, the football players are gradually reintegrated into football‐specific individual training or team training, so that more and more parts of a training session can be completed. The final parts of team training in ACL rehab are changes of direction in duels, body contact exercises and jumping exercises with the ball and other disruptive factors. As soon as full fitness for team training is possible and carried out, the incapacity to work ends for professional football players and a return to play testing should be carried out at this point at the latest [30]. Considerations include clinical assessment of knee stability, physical performance testing for functional performance, and subjective feedback from the athlete in confidence and readiness. These decisions are the last to be made by the medical team in football. From full team training until the decision for return to competition, only players and coaches are involved. The return to competition in professional football were published by a national ACL registry in football with a mean 247 days for professionals and mean 334 days for semiprofessional player and mean 376 days for amateur football players after ACL surgery. A return to competition after ACL‐re‐rupture was in professional player with 289 days more than 40 days later compared to primary ACL surgery. In amateur players was the return to competition after ACL‐re‐rupture with mean of 452 days more than 2.5 months later The surgical reconstruction of the ACL provides a successful return on football field, while after 3 years less than 50% of professional player and less than 25% of amateur football players hold their playing level or moving to upper playing level [43]. The high re‐rupture rate of ACL injuries in professional football of more than 17% [12], showed in the amateur football community with 20% higher rates [43]. Successful return to sports for football player's entails in all playing level should therefore address the unique demands of the sport depending on the players' performance level and experience. Each part of the return to sports process should be executed under supervision with knowledge in football‐specific movement patterns by professions like athletic coach, physiotherapist [19, 38]:

1) *Progressive return to football skills*
2) *High‐Intensity Interval Training (HIIT) for cardiovascular skills in football‐specific demands*
3) *Biomechanical analysis to receive football‐specific movement pattern and technique skills*
4) *Psychological readiness to achieve players' confidence and reduce fears for re‐injuries*

The decision‐making in the complete return to sports process is performed by the medical staff and the rehab coaches. Especially in the beginning of the treatment and rehab period of ACL injuries have decisions of ACL surgeons, medical doctors and physiotherapist priority. With the beginning of the individual and team training are also the rehabilitation and team coaches involved how return to sports steps were handled [38].

## SPECIFICS IN PROFESSIONAL FOOTBALL

The professional levels have a special status in all sports and require specific circumstances. Football in particular has built up very strong and broad professional structures worldwide. Considering that professional football players actually do their job full‐time and are among the most well paid athletes, the consequences of an ACL rupture are particularly significant in professional football [30]. Knowing that ACL ruptures have a very high rate of direct return to the field through surgical stabilisation of almost 100% in professional football [5], in the midterm after a few years there may be a reduction in physical abilities and sporting success in the affected professional player [6, 43]. It is also known that ACL ruptures are one of the most important reasons for ending a career in professional football and also for disability after the career [27]. Therefore, the characteristics of professional football include not only a high level of public attention for the sport and its players, but also a corresponding extrinsic and intrinsic pressure on injured players. The extrinsic pressure can be summed up on the one hand with the publicity, but also with the multiple influencing factors on professional football player, of which the influences of their own club, but also of agents are the most important. In addition, professional football players themselves are under intrinsic pressure to find the quickest possible way back onto the field after an injury. The sum of these influences in the management of serious injuries such as ACL rupture leads to players, their environment and, in some cases, medical professionals deviating from the standard of treatment. A narrative review from 2024 reports in a literature review and a summary of practical experiences from the daily routine of professional football from 16 different football‐specific situations of a professional football player with an acute ACL rupture, how and which pressure situations can arise. The temporal and intrinsic pressure begins with the selection of an MRI appointment, the search for an experienced surgeon, the selection of the right timing of the surgery or the typical acceleration of the rehabilitation processes. During the long timeout in football after an ACL rupture, professional players have to balance their external influences and their own wishes with regard to such pressure situations in such a way that, despite time pressure and the desire for a quick return, no complications or problems arise in the treatment of ACL ruptures [30].

## FUTURE OUTLOOKS AND LIMITATIONS

Epidemiological surveys like injury registries represent a crucial role in injury prevention in football and should be performed in the future both by football clubs and football associations [32]. Other scientific areas that are also important in ACL care and are still poorly researched are the prevention strategies of ACL re‐ruptures and the reasons for a lack of sustainability of playing ability after return to sports. Due to the long timeout that football players need after ACL ruptures to restore knee stability and their muscular and neuromotor abilities, prevention concepts are even more important and the scientifically available evidence‐based programmes must find their way from the literature into daily routine of amateur and professional football. In particular, the implementation of preventive exercises in everyday practice, in training and in the warm‐up before the game is still weak in football and must be brought into focus through improved knowledge transfer, especially in coach training. As a narrative review, this article does not provide scientific evidence in every aspect of the management of ACL ruptures in football, but it can present the current situation of basic sport‐specific procedures for the world's most important sport, football [40].

From a football perspective, and considering the expectations of players, clubs, and medical professionals, the very long downtime for footballers remains a particularly unresolved issue when considering the management of ACL ruptures. Therefore, future efforts should focus on shortening the rehabilitation phase after ACL surgery. This requires refining the currently used surgical techniques, improving rehabilitation efficiency, and improving secondary prevention.

## CONCLUSION

ACL ruptures occur frequently in football due to the sport‐specific stresses. While non‐surgical treatments for complete and unstable ACL ruptures have nearly no place in stop‐and‐go‐sport like football with its changes of direction and contact mechanism, surgical restoration of stability in the joint through ACL reconstruction shows very good results and therefore has the highest priority, especially in professional football. Football‐specific surgical technique for ACL injuries are widely discussed but differences in the management to other sports are not scientifically based.

## AUTHOR CONTRIBUTIONS

Considering that this manuscript is a narrative review, all authors contributed with a specific part in the preparation of the manuscript. Both main authors lead the group to the final version of the manuscript.

## CONFLICT OF INTEREST STATEMENT

The authors declare no conflicts of interest.

## ETHICS STATEMENT

Considering that this manuscript is a narrative review, no ethical approval is necessary here. Considering that this manuscript is a narrative review, no patient consent statement is necessary.

## Data Availability

Considering that this manuscript is a narrative review, no original data are provided here.

## References

[1] Achenbach L , Bloch H , Klein C , Damm T , Obinger M , Rudert M , et al. Four distinct patterns of anterior cruciate ligament injury in women's professional football (soccer): a systematic video analysis of 37 match injuries. Br J Sports Med. 2024;58(13):709–716. 38684328 10.1136/bjsports-2023-107113 PMC11228206

[2] Alentorn‐Geli E , Mendiguchía J , Samuelsson K , Musahl V , Karlsson J , Cugat R , et al. Prevention of non‐contact anterior cruciate ligament injuries in sports. Part II: systematic review of the effectiveness of prevention programmes in male athletes. Knee Surg Sports Traumatol Arthrosc. 2014;22(1):16–25. 24162718 10.1007/s00167-013-2739-x

[3] Alentorn‐Geli E , Myer GD , Silvers HJ , Samitier G , Romero D , Lázaro‐Haro C , et al. Prevention of non‐contact anterior cruciate ligament injuries in soccer players. Part 1: Mechanisms of injury and underlying risk factors. Knee Surg Sports Traumatol Arthrosc. 2009;17(7):705–729. 19452139 10.1007/s00167-009-0813-1

[4] Barengo N , Meneses‐Echávez J , Ramírez‐Vélez R , Cohen D , Tovar G , Bautista J . The impact of the FIFA 11+ training program on injury prevention in football players: a systematic review. Int J Environ Res Public Health. 2014;11(11):11986–12000. 25415209 10.3390/ijerph111111986 PMC4245655

[5] Bloch H , Reinsberger C , Klein C , Luig P , Krutsch W . High revision arthroscopy rate after ACL reconstruction in men's professional team sports. Knee Surg Sports Traumatol Arthrosc. 2023;31(1):142–151. 35976389 10.1007/s00167-022-07105-0

[6] Bonanzinga T , Grassi A , Altomare D , Lucidi GA , Macchiarola L , Zaffagnini S , et al. High return to sport rate and few re‐ruptures at long term in professional footballers after anterior cruciate ligament reconstruction with hamstrings. Knee Surg Sports Traumatol Arthrosc. 2022;30(11):3681–3688. 35451640 10.1007/s00167-022-06944-1

[7] Borque KA , Jones M , Laughlin MS , Balendra G , Willinger L , Pinheiro VH , et al. Effect of lateral extra‐articular tenodesis on the rate of revision anterior cruciate ligament reconstruction in elite athletes. Am J Sports Med. 2022;50(13):3487–3492. 36255290 10.1177/03635465221128828

[8] Bottoni CR , Smith EL , Shaha J , Shaha SS , Raybin SG , Tokish JM , et al. Autograft versus allograft anterior cruciate ligament reconstruction: a prospective, randomized clinical study with a minimum 10‐year follow‐up. Am J Sports Med. 2015;43(10):2501–2509. 26311445 10.1177/0363546515596406

[9] Crossley KM , Patterson BE , Culvenor AG , Bruder AM , Mosler AB , Mentiplay BF . Making football safer for women: a systematic review and meta‐analysis of injury prevention programmes in 11 773 female football (soccer) players. Br J Sports Med. 2020;54(18):1089–1098. 32253193 10.1136/bjsports-2019-101587 PMC7497572

[10] Crum RJ , de Sa D , Kanakamedala AC , Obioha OA , Lesniak BP , Musahl V . Aperture and suspensory fixation equally efficacious for quadriceps tendon graft fixation in primary ACL reconstruction: a systematic review. J Knee Surg. 2020;33(7):704–721. 30959537 10.1055/s-0039-1685160 PMC7683008

[11] Della Villa F , Buckthorpe M , Grassi A , Nabiuzzi A , Tosarelli F , Zaffagnini S , et al. Systematic video analysis of ACL injuries in professional male football (soccer): injury mechanisms, situational patterns and biomechanics study on 134 consecutive cases. Br J Sports Med. 2020;54(23):1423–1432. 32561515 10.1136/bjsports-2019-101247

[12] Della Villa F , Hägglund M , Della Villa S , Ekstrand J , Waldén M . High rate of second ACL injury following ACL reconstruction in male professional footballers: an updated longitudinal analysis from 118 players in the UEFA Elite Club Injury Study. Br J Sports Med. 2021;55(23):1350–1357. 33846157 10.1136/bjsports-2020-103555 PMC8606446

[13] Dowling AV , Corazza S , Chaudhari AMW , Andriacchi TP . Shoe‐surface friction influences movement strategies during a sidestep cutting task: implications for anterior cruciate ligament injury risk. Am J Sports Med. 2010;38(3):478–485. 20194954 10.1177/0363546509348374

[14] Ekeland A , Engebretsen L , Fenstad AM , Heir S . Similar risk of ACL graft revision for alpine skiers, football and handball players: the graft revision rate is influenced by age and graft choice. Br J Sports Med. 2020;54(1):33–37. 31399428 10.1136/bjsports-2018-100020

[15] Ekstrand J . Enceclopaedia of football medicine. Injury diagnosis and treatment. 2. Berlin: Thieme; 2017.

[16] Ekstrand J , Krutsch W , Spreco A , van Zoest W , Roberts C , Meyer T , et al. Time before return to play for the most common injuries in professional football: a 16‐year follow‐up of the UEFA Elite Club Injury Study. Br J Sports Med. 2020;54(7):421–426. 31182429 10.1136/bjsports-2019-100666 PMC7146935

[17] Farinelli L , Abermann E , Meena A , Ueblacker P , Hahne J , Fink C . Return to play and pattern of injury after ACL rupture in a consecutive series of elite UEFA soccer players. Orthop J Sports Med. 2023;11(3):23259671231153629. 10.1177/23259671231153629 36896098 PMC9989402

[18] Farinelli L , Csapo R , Meena A , Abermann E , Hoser C , Fink C . Concomitant injuries associated with ACL rupture in elite professional alpine ski racers and soccer players: a comparative study with propensity score matching analysis. Orthop J Sports Med. 2023;11(8):23259671231192127. 10.1177/23259671231192127 37655251 PMC10467387

[19] Figueroa D , Arce G , Espregueira‐Mendes J , Maestu R , Mosquera M , Williams A , et al. Return to sport soccer after anterior cruciate ligament reconstruction: ISAKOS consensus. J ISAKOS. 2022;7(6):150–161. 35998884 10.1016/j.jisako.2022.08.004

[20] Gföller P , Abermann E , Runer A , Hoser C , Pflüglmayer M , Wierer G , et al. Non‐operative treatment of ACL injury is associated with opposing subjective and objective outcomes over 20 years of follow‐up. Knee Surg Sports Traumatol Arthrosc. 2019;27(8):2665–2671. 30467579 10.1007/s00167-018-5296-5

[21] Gokeler A , Tosarelli F , Buckthorpe M , Della Villa F . Neurocognitive errors and noncontact anterior cruciate ligament injuries in professional male soccer players. J Athl Train. 2024;59(3):262–269. 37248515 10.4085/1062-6050-0209.22 PMC10976343

[22] Gordon J , Nordström A . Trauma and medical emergencies. Encyclopedia of football medicine. 1. Stuttgart: Thieme; 2017.

[23] Grassi A , Macchiarola L , Filippini M , Lucidi GA , Della Villa F , Zaffagnini S . Epidemiology of anterior cruciate ligament injury in italian first division soccer players. Sports Health. 2020;12(3):279–288. 31800358 10.1177/1941738119885642 PMC7222666

[24] Herbawi F , Lozano‐Lozano M , Lopez‐Garzon M , Postigo‐Martin P , Ortiz‐Comino L , Martin‐Alguacil JL , et al. A systematic review and meta‐analysis of strength recovery measured by isokinetic dynamometer technology after anterior cruciate ligament reconstruction using quadriceps tendon autografts vs. hamstring tendon autografts or patellar tendon autografts. Int J Environ Res Public Health. 2022;19(11):6764. 35682357 10.3390/ijerph19116764 PMC9180841

[25] Hildebrandt C , Müller L , Zisch B , Huber R , Fink C , Raschner C . Functional assessments for decision‐making regarding return to sports following ACL reconstruction. Part I: development of a new test battery. Knee Surg Sports Traumatol Arthrosc. 2015;23(5):1273–1281. 25682164 10.1007/s00167-015-3529-4 PMC4555192

[26] Jader A , Melo Cué RJ , Romandini I , Zikria BA , Papakostas E , Marín Fermín T . Injection therapy in professional footballers. Int Orthop. 2024;48(11):2827–2834. 39283321 10.1007/s00264-024-06301-6

[27] Koch M , Klügl M , Frankewycz B , Lang S , Worlicek M , Popp D , et al. Football‐related injuries are the major reason for the career end of professional male football players. Knee Surg Sports Traumatol Arthrosc. 2021;29(11):3560–3568. 34370085 10.1007/s00167-021-06684-8 PMC8514381

[28] Koga H , Bahr R , Myklebust G , Engebretsen L , Grund T , Krosshaug T . Estimating anterior tibial translation from model‐based image‐matching of a noncontact anterior cruciate ligament injury in professional football: a case report. Clin J Sport Med. 2011;21(3):271–274. 21487293 10.1097/JSM.0b013e31821899ec

[29] Krause M , Freudenthaler F , Frosch KH , Achtnich A , Petersen W , Akoto R . Operative versus conservative treatment of anterior cruciate ligament rupture. Dtsch Arztebl Int. 2018;115(51–52):855–862. 30765021 10.3238/arztebl.2018.0855 PMC6381773

[30] Krutsch W , Kobes T , Huber L , Szymski D , Geßlein M , Rüther J , et al. [Complex knee injuries in football: management from injury to return to competition]. Orthopadie. 2024;53(6):438–448. 38801525 10.1007/s00132-024-04508-4

[31] Krutsch W , Lehmann J , Jansen P , Angele P , Fellner B , Achenbach L , et al. Prevention of severe knee injuries in men's elite football by implementing specific training modules. Knee Surg Sports Traumatol Arthrosc. 2020;28(2):519–527. 31541292 10.1007/s00167-019-05706-w

[32] Krutsch W , Loose O . Gesamtkonzept zur Verletzungsprävention von schweren Kniegelenksverletzungen im Leistungsfußball. Arthroskopie: Springer Berlin Heidelberg; 2020.

[33] Krutsch W , Zeman F , Zellner J , Pfeifer C , Nerlich M , Angele P . Increase in ACL and PCL injuries after implementation of a new professional football league. Knee Surg Sports Traumatol Arthrosc. 2016;24(7):2271–2279. 25293676 10.1007/s00167-014-3357-y

[34] Kunze KN , Moran J , Polce EM , Pareek A , Strickland SM , Williams RJ . Lower donor site morbidity with hamstring and quadriceps tendon autograft compared with bone‐patellar tendon‐bone autograft after anterior cruciate ligament reconstruction: a systematic review and network meta‐analysis of randomized controlled trials. Knee Surg Sports Traumatol Arthrosc. 2023;31(8):3339–3352. 37000243 10.1007/s00167-023-07402-2

[35] Manojlovic M , Ninkovic S , Matic R , Versic S , Modric T , Sekulic D , et al. Return to play and performance after anterior cruciate ligament reconstruction in soccer players: a systematic review of recent evidence. Sports Med. 2024;54(8):2097–2108. 38710914 10.1007/s40279-024-02035-y PMC11329701

[36] Memmel C , Krutsch W , Szymski D , Pfeifer C , Henssler L , Frankewycz B , et al. Current standards of early rehabilitation after anterior cruciate ligament reconstruction in German speaking countries‐differentiation based on tendon graft and concomitant injuries. Int J Environ Res Public Health. 2022;19(7):4060. 35409745 10.3390/ijerph19074060 PMC8997891

[37] Montalvo AM , Schneider DK , Silva PL , Yut L , Webster KE , Riley MA , et al. ‘What's my risk of sustaining an ACL injury while playing football (soccer)?’ A systematic review with meta‐analysis. Br J Sports Med. 2019;53(21):1333–1340. 29599121 10.1136/bjsports-2016-097261 PMC6642026

[38] Musahl V , Karlsson J , Krutsch W , Mandelbaum BR , Espregueira‐Mendes J , d'Hooge P . Return to play in football – an evidence‐based approach. Berlin Heidelberg: Springer; 2018.

[39] Pinheiro VH , Borque KA , Laughlin MS , Jones M , Balendra G , Kent MR , et al. Determinants of performance in professional soccer players at 2 and 5 years after ACL reconstruction. Am J Sports Med. 2023;51(14):3649–3657. 37960868 10.1177/03635465231207832

[40] Prill R , Królikowska A , Enes Kayaalp M , Ramadanov N , Karlsson J , Hirschmann MT . Enhancing research methods: The role of systematic and scoping reviews in orthopaedics, sports medicine and rehabilitation. J Exp Orthop. 2024;11(4):e70069. 10.1002/jeo2.70069 39502322 PMC11534858

[41] Sandon A , Krutsch W , Alt V , Forssblad M . Increased occurrence of ACL injuries for football players in teams changing coach and for players going to a higher division. Knee Surg Sports Traumatol Arthrosc. 2022;30(4):1380–1387. 33987689 10.1007/s00167-021-06604-w PMC9007801

[42] Silvers‐Granelli HJ , Bizzini M , Arundale A , Mandelbaum BR , Snyder‐Mackler L . Does the FIFA 11+ injury prevention program reduce the incidence of ACL injury in male soccer players? Clin Orthop Relat Res. 2017;475(10):2447–2455. 28389864 10.1007/s11999-017-5342-5 PMC5599387

[43] Szymski D , Achenbach L , Weber J , Huber L , Memmel C , Kerschbaum M , et al. Reduced performance after return to competition in ACL injuries: an analysis on return to competition in the ‘ACL registry in German Football’. Knee Surg Sports Traumatol Arthrosc. 2023;31(1):133–141. 35819462 10.1007/s00167-022-07062-8 PMC9859836

[44] Szymski D , Achenbach L , Zellner J , Weber J , Koch M , Zeman F , et al. Higher risk of ACL rupture in amateur football compared to professional football: 5‐year results of the ‘Anterior cruciate ligament‐registry in German football’. Knee Surg Sports Traumatol Arthrosc. 2022;30(5):1776–1785. 34524500 10.1007/s00167-021-06737-y PMC9033691

[45] Tischer T , Karlsson J , Seil R . Sport‐specific differences in ACL injury, treatment and return to sports. Knee Surg Sports Traumatol Arthrosc. 2024;32(6):1359–1362. 38586977 10.1002/ksa.12174

[46] van Melick N , van Cingel REH , Brooijmans F , Neeter C , van Tienen T , Hullegie W , et al. Evidence‐based clinical practice update: practice guidelines for anterior cruciate ligament rehabilitation based on a systematic review and multidisciplinary consensus. Br J Sports Med. 2016;50(24):1506–1515. 27539507 10.1136/bjsports-2015-095898

[47] Waldén M , Hägglund M , Magnusson H , Ekstrand J . Anterior cruciate ligament injury in elite football: a prospective three‐cohort study. Knee Surg Sports Traumatol Arthrosc. 2011;19(1):11–19. 20532869 10.1007/s00167-010-1170-9

[48] Xu B , Yin Y , Zhu Y , Yin Y , Fu W . Comparison of bioabsorbable and metallic interference screws for graft fixation during ACL reconstruction: a meta‐analysis of randomized controlled trials. Orthop J Sports Med. 2021;9(8):23259671211021577. 10.1177/23259671211021577 34423056 PMC8377324

[49] Xu D , Zhou H , Wang M , Ma X , Gusztav F , Chon TE , et al. Contribution of ankle motion pattern during landing to reduce the knee‐related injury risk. Comput Biol Med. 2024;180:108965. 10.1016/j.compbiomed.2024.108965 39084051

